# Sarcopenia Transitions and Influencing Factors Among Chinese Older Adults With Multistate Markov Model

**DOI:** 10.1093/geroni/igad105

**Published:** 2023-09-20

**Authors:** Boran Sun, Shatao Li, Yanbo Wang, Wenbo Xiao, Han Zhao, Xuewei Liu, Yang Liu, Xinlin Lu, Bei Gao, Jiangtao Zhou, Bingyi Wang, Yuan Wang, Yongjie Chen, Wenli Lu

**Affiliations:** Department of Epidemiology and Statistics, School of Public Health, Tianjin Medical University, Tianjin, People’s Republic of China; Department of Epidemiology and Statistics, School of Public Health, Tianjin Medical University, Tianjin, People’s Republic of China; Department of Epidemiology and Statistics, School of Public Health, Tianjin Medical University, Tianjin, People’s Republic of China; Department of Epidemiology and Statistics, School of Public Health, Tianjin Medical University, Tianjin, People’s Republic of China; Department of Epidemiology and Statistics, School of Public Health, Tianjin Medical University, Tianjin, People’s Republic of China; Department of Epidemiology and Statistics, School of Public Health, Tianjin Medical University, Tianjin, People’s Republic of China; Department of Epidemiology and Statistics, School of Public Health, Tianjin Medical University, Tianjin, People’s Republic of China; Department of Epidemiology and Statistics, School of Public Health, Tianjin Medical University, Tianjin, People’s Republic of China; Department of Epidemiology and Statistics, School of Public Health, Tianjin Medical University, Tianjin, People’s Republic of China; Department of Epidemiology and Statistics, School of Public Health, Tianjin Medical University, Tianjin, People’s Republic of China; Department of Epidemiology and Statistics, School of Public Health, Tianjin Medical University, Tianjin, People’s Republic of China; Department of Epidemiology and Statistics, School of Public Health, Tianjin Medical University, Tianjin, People’s Republic of China; Department of Epidemiology and Statistics, School of Public Health, Tianjin Medical University, Tianjin, People’s Republic of China; Department of Epidemiology and Statistics, School of Public Health, Tianjin Medical University, Tianjin, People’s Republic of China

**Keywords:** Multistate Markov model, Possible sarcopenia, Sarcopenia, Transitions

## Abstract

**Background and Objectives:**

Little is known about the sarcopenia transition process across different stages among Chinese community-dwelling older adults. We aimed to explore dynamic transitions of sarcopenia and its influencing factors in Chinese older adults.

**Research Design and Methods:**

Data were derived from the China Health and Retirement Longitudinal Study. A total of 2856 older adults with complete data in the 2011, 2013, and 2015 waves were included in our study. Participants were categorized into 3 states: no sarcopenia, possible sarcopenia, and sarcopenia according to the Asian Working Group for Sarcopenia 2019 (AWGS 2019) criteria. Continuous-time multistate Markov model was performed to estimate the 1-year transition probabilities and the associated factors of sarcopenia transitions. The association strength was expressed as hazard ratio and 95% confidence interval.

**Results:**

The progression and reversion rates between no sarcopenia and sarcopenia state were 6.01 and 9.20 per 100 person-years, respectively. The 1-year progression probability to possible sarcopenia was higher compared with the likelihood of moving to the sarcopenia state (0.127 vs 0.034). The reversion probability to no sarcopenia was also higher among those with possible sarcopenia (0.281 vs 0.157). Older age, rural living, worse cognition status, higher chronic disease numbers, and lower nutrition status measured by body mass index accelerated the sarcopenia progression. Cognition status and body mass index level were related to higher chances of reverting.

**Discussion and Implications:**

Possible sarcopenia might be a critical time window to promote sarcopenia reversion. Timely interventions aimed at delaying the progression and facilitating sarcopenia recovery should focus on improving cognitive function and nutrition levels.

**Translational Significance**: It is not clear about the sarcopenia transitions and its influencing factors among Chinese community-dwelling older adults. Our findings indicated that identifying older adults with possible sarcopenia was of great importance for early prevention and reducing the burdens of sarcopenia. Targeted nutritional and cognitive interventions should be implemented for healthy and sarcopenic older adults to delay the progression of sarcopenia and promote its rehabilitation. Recognizing the critical time window and effective interventions for sarcopenia prevention could contribute to achieving healthy aging in China.

It is estimated that the number of people aged 65 and older will exceed 1.6 billion by 2050, accounting for more than 16% of the global population (1). With the global aging process accelerating, sarcopenia has been a major public health concern among older adults with a projection of 200 million people prevalent with sarcopenia worldwide by 2050 (2). Sarcopenia is a geriatric syndrome with a hallmark of age-related progressive skeletal muscle disorder, involving the accelerated loss of muscle mass and function (3). Recently, considerable studies have illustrated that sarcopenia was associated with increased mental and physical adverse outcomes including depression, falls, cardiovascular diseases, and mortality (4–8). Thus, it is noteworthy to explore means to delay its occurrence and progression among older adults.

Up till now, different criteria have been proposed to identify sarcopenia based on both the loss of muscle mass and muscle function. The European Working Group on Sarcopenia in Older People (EWGSOP) proposed and updated the diagnostic algorithms of sarcopenia for European older adults in 2010 (EWGSOP 1) and 2018 (EWGSOP 2) (9,10), which classified older adults into 4 categories: no sarcopenia, probable sarcopenia, nonsevere sarcopenia, and severe sarcopenia. Considering differences in anthropometric and lifestyle compared with the western aged, the Asian Working Group for Sarcopenia (AWGS) proposed the AWGS 2014 consensus and updated it in 2019 with growing research evidence in Asia. It was worth noting that the AWGS 2019 considered muscle mass as a primary diagnostic indicator rather than muscle strength and revised the cutoffs of muscle strength and physical performance based on pooled evidence of previous Asian studies. In addition, possible sarcopenia, defined by either low muscle strength or reduced physical performance only, was first introduced in Asians for the purpose of early detection and implementing lifestyle interventions in primary health care (11).

The nature of the dynamic development of sarcopenia has been revealed regardless of based on the EWGSOP or the AWGS diagnostic algorithm. In the past decades, it has increasingly been recognized that dynamic transitions exist between different sarcopenia statuses. Murphy and his colleagues found that the process of sarcopenia transitions was dynamic and moderate physical exercises contributed to increasing the probability of transitioning to no sarcopenia among Americans (12). A population-based study in Sweden also revealed that 2-way transitions might exist for the earliest sarcopenia status and factors including physical activity, cognitive function, and chronic conditions were identified to delay sarcopenia progression (13). However, to our knowledge, no studies have ever applied the AWGS 2019 criteria to conduct research on the sarcopenia transitions and influencing factors, especially for Chinese community-dwelling older adults.

To fill this gap, we aimed to explore transitions across sarcopenia status according to the AWGS 2019 and to identify factors associated with transitions among Chinese older adults.

## Method

### Study Design and Population

The China Health and Retirement Longitudinal Study (CHARLS) was a nationally representative longitudinal survey among people aged 45 years and older in China, with the baseline survey starting in 2011. All participants were followed every 2 years since baseline by using a face-to-face personal interview and standardized questionnaires by trained investigators were used to collect data on sociodemographic, lifestyle factors, and health status. Death information was obtained from registrations and certifications by asking the relatives or local communities of the deceased. The details have been published in previous studies (14).

**Figure 1.**
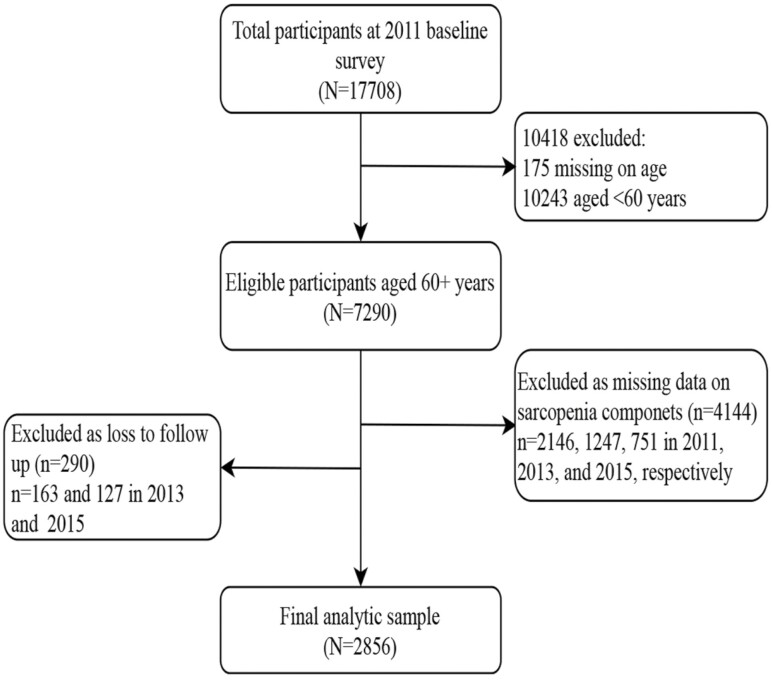
Flowchart of the analytic sample.

In the study, data were derived from the CHARLS in the 2011, 2013, and 2015 waves. The inclusion criteria were individuals aged at least 60 years old and having data to define sarcopenia status. Older adults with loss to follow-up were excluded. A total of 2856 participants were included as the final analytic sample. The detailed sample selection process is shown in Figure 1. The study was approved by the Ethical Review Committee of Peking University (IRB00001052-11015).

### Assessment of Sarcopenia Status

Sarcopenia status was assessed based on the AWGS 2019 diagnostic algorithm, including muscle mass, muscle strength, and physical performance (11). First, muscle mass was measured by the appendicular skeletal muscle mass (ASM) using a reliable and validated anthropometric equation for the Chinese population as follows: ASM = 0.193 * weight + 0.107 * height – 4.157 * sex – 0.037 * age – 2.631 (15). Weight (kg) and height (cm) were measured using a digital scale and stadiometer, respectively. The value 1 of sex represented men and the value 2 of sex was women. As previously described, the cutoff value for low muscle mass was determined based on the sex-specific lowest 20% of the height-adjusted muscle mass (ASM/Ht^2^) (16,17). In our study, ASM/Ht^2^ values of <6.79kg/m^2^ for males and <4.93 kg/m^2^ for females were defined as low muscle mass.

Secondmuscle strength was measured by handgrip strength (11). In the CHARLS, participants were asked to stand and hold the dynamometer (Nantong Yuejian TM WL-1000 dynamometer) at a right angle and squeeze with maximum effort twice for each hand. The maximum value of 4 measurements was used for statistical analysis (18). Low muscle strength was defined as the handgrip strength <28 kg in men and <18 kg in women. In line with previous studies, handgrip strength was considered as missing in the case of not using full effort, or outlier data (<1 percentile or >99 percentile) (19).

Third, a short physical performance battery score (SPPB) test was applied to assess physical performance. SPPB consisted of the 2.5-m walking test, repeated the chair-standing test, and balance test (11). Each test was assigned a score ranging from 0 to 4 and the total score ranged from 0 to 12 (20–22). SPPB ≤9 was defined to have a low physical performance (11). For the walking test, participants were asked to walk on a 2.5-m course at a normal pace twice to obtain walking speed based on the recorded seconds (23). The faster speed of the 2 tests was used for statistical analysis. Scores were assigned as follows: unable to complete (0 point), ≤0.43 m/s (1 point), 0.44–0.60 m/s (2 points), 0.61–0.77 m/s (3 points), and ≥0.78 m/s (4 points) (24). For the repeated chair-standing test, participants were asked to stand up straight and then sit down 5× as fast as they could without using their arms to push themselves off the chair. Scores were based on total seconds to complete the 5 stands and were assigned as follows: unable to perform (0 point), ≥16.7 seconds (1 point), 13.7–16.6 seconds (2 points), 11.2–13.6 seconds (3 points), and ≤11.1 seconds (4 points) (24). As for the balance test, participants were initially asked to perform the semitandem stand test. Participants unable to hold for 10 seconds were asked to perform the side-by-side stand for 10 seconds. While participants completing this position carried on the full-tandem stand test for the 30 s or 60 s. Scores were as follows: unable to perform (0 point), side-by-side for 10 seconds and semitandem <10 seconds (1 point), semitandem for 10 seconds and full-tandem 0–2 seconds (2 points), full-tandem 3–9 seconds (3 points), full-tandem 10 seconds or longer (4 points) (24).

According to the AWGS 2019, possible sarcopenia was defined by normal muscle mass and low muscle strength or physical performance. Sarcopenia was diagnosed when low muscle mass plus low muscle strength or low physical performance were detected. When low muscle strength, low muscle mass, and low physical performance were all present, severe sarcopenia was defined. Participants without any low muscle strength, low muscle mass, and low physical performance were classified as having no sarcopenia (11). In the final analytic sample, 92 (3.2%) participants were classified as the severe sarcopenia status. Thus, subjects with severe sarcopenia were merged into the sarcopenia group.

### Covariates

The covariates included in our study are possible predictors related to sarcopenia and involved 3 categories, namely sociodemographic factors, health-related factors, and behavior-risk factors in the CHARLS (14). Sociodemographic factors included age (60–74 vs ≥75), sex (male vs female), region (rural vs urban), and education level. Due to the general low education level among middle-aged and older Chinese, education level was categorized into lower than elementary school and elementary school or above (25). Health-related factors contained difficulties in the basic activity of daily living (ADL), cognition function, and the number of chronic diseases. ADL difficulties included any difficulties in performing dressing, bathing, feeding, moving from bed to chair, using the toilet, and maintaining continence (26). Cognitive function was assessed through face-to-face interviews by trained investigators. The total cognition score was calculated by summing the domains of orientation, attention, and calculation, visuospatial ability, and episodic memory and ranged from 0 to 30. A higher score was an indicator of better cognitive function (27). The total number of chronic diseases was calculated as the sum of specific medical conditions as follows: hypertension, diabetes, dyslipidemia, stroke, asthma, arthritis or rheumatism, heart disease, chronic lung diseases, kidney diseases, liver disease, stomach or other digestive disease, cancer, memory problems and emotional, nervous, or psychiatric problems (14). While behavior-risk factors included habits of smoking and drinking (current vs noncurrent) and nutritional status (measured by body mass index (BMI)).

### Statistical Analysis

Continuous variables with normal distribution were presented as means and standard deviation (*SD*). Non-normal variables were reported as median and interquartile range (IQR), whereas categorical variables were expressed as frequencies (percentages). Baseline characteristics across sarcopenia groups and between the analytic sample and older participants excluded with missing sarcopenia data were compared using 2 independent sample *t* test, *analysis of variance*, *chi-square*, Mann–Whitney *U*, and *Kruskal*–*Wallis* test, as appropriate.

Continuous-time multistate Markov model without including covariates was first employed to estimate transition intensities, 1-year transition probabilities, and the mean permanence time in each status (28). The continuous-time multistate Markov model was used to describe a process in which an individual moves through states in continuous time, which had assumptions as follows: transition intensities remained constant over time, and the future state depended on the current state and was independent of historical states (29). In our study, mutual transitions were allowed between different sarcopenia statuses and the time scale was the follow-up time (years) since the baseline wave. Death was modeled as an absorbing state and we considered other states as transient states. Similar to other longitudinal studies using the multistate Markov model (28,29), we constructed the multistate Markov model using the R package msm, which allowed for fitting data from processes with arbitrary observation times and had robust estimations without prior assumption of exact transition time in the panel data. To improve the convergence of the model, the quasi-Newton optimization algorithm was employed (30). Influential factors through sarcopenia transitions were analyzed using proportional intensity models. The effects of covariates on each transition were expressed as hazard ratios with 95% confidence intervals (CIs). The values at the beginning of each observed transition were introduced into the model to analyze the effects of time-varying covariates such as cognition and the number of chronic diseases on each transition. Details have been published in previous studies (13,31,32).

Sensitivity analysis was first performed to include participants with loss to follow-up. Death and loss to follow up were both considered as absorbing states. The transition intensities and probabilities were reevaluated and differences were compared with primary results. Additionally, we checked for the differences between men and women in the transition probabilities and associated factors through sarcopenia transitions. Analyses were performed using R version 4.1.1 (R Foundation for Statistical Computing). A 2-tailed *p* ≤ .05 indicated a statistical significance.

## Results

### Characteristic of Participants

A total of 2856 eligible participants were enrolled as the final analytic sample (Figure 1). The number of participants for no sarcopenia, possible sarcopenia, and sarcopenia were 1 505, 1 019, and 332, respectively. The prevalence of possible sarcopenia and sarcopenia was 35.7% (1 019/2 856) and 11.6% (332/2 856), respectively. The mean (SD) age of the included sample was 67.6 (6.1) years, and 1 383 (48.4%) were females. The sarcopenia prevalence in females was higher (12.4%) compared with males (10.9%). Participants with sarcopenia were more likely to be older females, less educated, having a higher prevalence of ADL difficulties, a lower cognition level, and a higher number of chronic diseases than those with sarcopenia-free (all *p* < .001). Moreover, older adults with sarcopenia were more likely to be noncurrent smokers and drinkers and to have a lower BMI level (Table 1). Compared with older adults with missing sarcopenia information, the analytic sample was more likely to be younger, living in rural areas, having less ADL difficulties, and being current smokers (Supplementary Table 1).

**Table 1.** Baseline Characteristics of Participants by Sarcopenia Status

Characteristics	All	No Sarcopenia	Possible Sarcopenia	Sarcopenia	*p*
*N* (%)	2 856 (100.0)	1 505 (52.7)	1 019 (35.7)	332 (11.6)	
*Sociodemographic factors*					
Age (y), mean (*SD*)^*^	67.6 (6.1)	66.1 (5.1)	68.1 (6.2)	73.1 (6.8)	<.001
60–74 y old, *n* (%)^†^	2 416 (84.6)	1 389 (92.3)	847 (83.1)	180 (54.2)	<.001
Female, *n* (%)^†^	1 383 (48.4)	673 (44.7)	539 (52.9)	171 (51.5)	<.001
Rural living, *n* (%)^†^	1 992 (69.7)	1 034 (68.7)	685 (67.2)	273 (82.2)	<.001
Elementary school or above, *n* (%)^†^	1 177 (41.2)	737 (49.0)	368 (36.1)	72 (21.7)	<.001
*Health-related factor* **s**					
ADL difficulty, *n* (%)^†^	570 (20.1)	207 (13.9)	274 (27.1)	89 (27.1)	<.001
Cognition score, mean (*SD*) ^*^	12.6 (5.7)	13.7 (5.4)	11.8 (5.6)	9.5 (5.6)	<.001
Number of chronic diseases, median (IQR) ^‡^	1.0 (0.0, 2.0)	1.0 (0.0, 2.0)	2.0 (1.0, 3.0)	1.0 (0.0, 2.0)	<.001
*Behavior-risk factors*					
Current smokers, *n* (%)^†^	937 (33.0)	523 (34.8)	302 (29.8)	112 (34.6)	.028
Current drinkers, *n* (%)^†^	861 (30.2)	511 (34.0)	267 (26.3)	83 (25.1)	<.001
BMI (kg/m^2^), mean (*SD*)^*^	22.7 (3.8)	22.8 (3.7)	23.9 (3.5)	18.3 (1.7)	<.001
Handgrip strength (kg), mean (*SD*)^*^	29.2 (8.5)	32.2 (7.4)	26.6 (8.6)	23.7 (7.3)	<.001
Low muscle strength, *n* (%)^†^	484 (16.9)	0 (0.0)	327 (32.1)	157 (47.3)	<.001
ASM/Ht^2^ (kg/m^2^), mean (*SD*)^*^	6.5 (1.1)	6.6 (1.1)	6.7 (1.0)	5.5 (1.1)	<.001
Low muscle mass, *n* (%)^†^	587 (20.6)	255 (16.9)	0 (0.0)	332 (100.0)	<.001
SPPB score, mean (*SD*) ^*^	9.4 (2.5)	11.1 (0.8)	7.5 (2.5)	7.6 (2.7)	<.001
Low physical performance, *n* (%)^†^	1 176 (41.2)	0 (0.0)	909 (89.2)	267 (81.4)	<.001

*Notes*: ADL = activity of daily living; BMI = body mass index; ASM/Ht^2^ = height-adjusted appendicular skeletal muscle mass; *SD* = standard deviation; IQR = interquartile range; 24, 115, 2, 17, 6, and 1 missing for ADL, cognition, diseases numbers, smoking, drinking, and BMI.

^*^ These variables were compared using analysis of variance.

^†^These variables were compared using the chi-square test.

^‡^These variables were compared using the Kruskal–Wallis test.

### Transitions of Sarcopenia Status

A total of 5514 transitions occurred at follow-up checkups. In total 24.7% showed deterioration from no sarcopenia to possible sarcopenia and sarcopenia state at the following visits; 41.5% and 33.8% showed an improvement from the state of possible sarcopenia and sarcopenia, respectively (Table 2 and Supplementary Figure 1).

**Table 2.** Total Number and Percentages of Observed Sarcopenia Transitions During Follow up

To	From		
	No Sarcopenia (*n* = 3 062)	Possible Sarcopenia (*n* = 1 840)	Sarcopenia (*n* = 612)
	*N* (%)	*N* (%)	*N* (%)
No sarcopenia	2 202 (71.9)	763 (41.5)	154 (25.1)
Possible sarcopenia	584 (19.1)	859 (46.7)	53 (8.7)
Sarcopenia	173 (5.6)	82 (4.5)	317 (51.8)
Death	103 (3.4)	136 (7.3)	88 (14.4)

*Note*: Individuals could have experienced more than one transition.

During 10 825 person-years of follow up, 327 deaths occurred and the death rate was 1.85, 3.58, and 6.99 per 100 follow up person-years for older adults with no sarcopenia, possible sarcopenia, and sarcopenia, respectively. 23.3% of participants with no sarcopenia progressed to possible sarcopenia or sarcopenia state, with an incidence rate of 6.01/100 person-years. For those with possible sarcopenia, 5.1% progressed to the sarcopenia state. The progression rate was 1.36/100 person-years. While the regression rate was 11.47/100 and 9.20/100 person-years for older adults with possible sarcopenia and sarcopenia, respectively.

### Transition Intensities and 1-Year Probabilities

From the estimated transition intensities, the transition from no sarcopenia to possible sarcopenia was 4.1× as likely to transfer from no sarcopenia to sarcopenia. The likelihood of moving from possible sarcopenia to no sarcopenia was approximately twice as high as moving from sarcopenia to no sarcopenia. While for transition probabilities, no sarcopenia individuals had an 82.3% probability of remaining at year 1. And they were more likely to progress to the possible sarcopenia state than the sarcopenia state (12.7% vs 3.4%). Those with possible sarcopenia had a 65.4% probability of maintaining it. Sarcopenia older adults had a 71.5% probability of staying there, and the probability of reverting to no sarcopenia was lower than individuals with possible sarcopenia (15.7% vs 28.1%). Among the 3 groups, those with sarcopenia had the highest death probability, followed by possible sarcopenia, and no sarcopenia (Table 3).

**Table 3.** Estimated Transition Intensities and 1-Year Probabilities

Transition	Intensities (95% CI)	1-Year Probabilities (95% CI)
*From no sarcopenia*		
No sarcopenia	–	0.823 (0.810, 0.834)
Possible sarcopenia	0.176 (0.159, 0.194)	0.127 (0.117, 0.138)
Sarcopenia	0.043 (0.035, 0.051)	0.034 (0.029, 0.040)
Death	0.013 (0.009, 0.017)	0.016 (0.014, 0.020)
*From possible sarcopenia*		
Possible sarcopenia	–	0.654 (0.633, 0.672)
No sarcopenia	0.388 (0.356, 0.424)	0.281 (0.262, 0.300)
Sarcopenia	0.028 (0.019, 0.042)	0.025 (0.020, 0.034)
Death	0.045 (0.037, 0.055)	0.040 (0.035, 0.048)
*From sarcopenia*		
Sarcopenia	–	0.715 (0.686, 0.738)
No sarcopenia	0.199 (0.163, 0.242)	0.157 (0.135, 0.182)
Possible sarcopenia	0.053 (0.031, 0.090)	0.051 (0.037, 0.073)
Death	0.089 (0.071, 0.111)	0.077 (0.063, 0.095)

### Sojourn Time and Model Fit

The permanence time was shorter for the possible sarcopenia state (2.17 [95% CI: 2.00–2.33]), compared with the state of sarcopenia (2.94 [95% CI: 2.60–3.27]) and no sarcopenia (4.34 [95% CI: 3.99–4.70; Supplementary Table 2]). Moderate goodness of fit for the model without covariates is shown in Supplementary Figure 2.

### Influencing Factors of Sarcopenia Transitions

Table 4 presents the factors related to sarcopenia transitions (univariate analysis is shown in Supplementary Table 3). For sarcopenia-free older adults, factors associated with progression were advanced age, rural living, worse cognition status, a higher number of chronic diseases, and a lower BMI level. For those with possible sarcopenia, older age, rural living, noncurrent drinker, and a lower BMI level was associated with a higher probability of the development of sarcopenia. While the chances of reverting to no sarcopenia decreased with older age, a worse cognition status, and a lower BMI level. For participants with sarcopenia, the chance of reverting to a milder sarcopenia state was positively associated with being female and having better cognition status. Older males with possible sarcopenia and sarcopenia had a higher mortality risk. Sarcopenia-free older adults had a higher mortality risk with the increasing numbers of chronic diseases (Supplementary Table 4).

**Table 4.** Multivariate Analysis of Factors Associated with Sarcopenia Transitions (HR [95% CI])

Characteristics	From No Sarcopenia to		From Possible Sarcopenia to		From Sarcopenia to	
	Possible Sarcopenia	Sarcopenia	No Sarcopenia	Sarcopenia	No Sarcopenia	Possible Sarcopenia
*Sociodemographic factors*						
Age (ref = 60–74)	**1.59 (1.15, 2.21)**	**2.97 (1.29, 6.84)**	**0.32 (0.21, 0.47)**	**2.23 (1.16, 4.31)**	1.34 (0.68, 2.61)	0.50 (0.12, 2.08)
Sex (ref = male)	1.06 (0.80, 1.42)	1.69 (0.74, 3.90)	1.13 (0.87, 1.45)	0.41 (0.17, 1.00)	**5.23 (2.15, 12.76)**	0.22 (0.04, 1.59)
Region (ref = urban)	1.01 (0.77, 1.27)	**3.42 (1.05, 11.10)**	0.92 (0.74, 1.14)	**1.39 (1.19, 1.81)**	1.44 (0.67, 3.07)	0.48 (0.14, 1.63)
Education level (ref = lower than elementary school)	0.83 (0.64, 1.08)	1.62 (0.72, 3.64)	1.04 (0.83, 1.31)	0.35 (0.11, 1.14)	2.74 (1.42, 5.29)	0.41 (0.11, 1.56)
*Health-related factors*						
ADL difficulties (ref = no)	1.02 (0.78, 1.33)	1.53 (0.74, 3.18)	0.56 (0.44, 0.72)	0.75 (0.37, 1.51)	1.13 (0.63, 2.04)	0.93 (0.31, 2.81)
Cognition score (per 1-point increase)	**0.97 (0.95, 0.99)**	0.99 (0.94, 1.05)	**1.04 (1.02, 1.06)**	0.96 (0.89, 1.03)	**1.05 (1.01, 1.10)**	1.09 (0.99, 1.21)
Number of chronic diseases	**1.12 (1.04, 1.20)**	1.10 (0.89, 1.35)	0.97 (0.91, 1.03)	0.94 (0.77, 1.15)	1.05 (0.88, 1.25)	0.82 (0.56, 1.20)
*Behavior-risk factors*						
Smoking status (ref = noncurrent)	1.01 (0.77, 1.34)	1.01 (0.51, 1.98)	0.98 (0.76, 1.26)	1.07 (0.48, 2.41)	1.91 (0.97, 3.76)	0.74 (0.24, 2.31)
Drinking status (ref = noncurrent)	1.02 (0.78, 1.33)	1.15 (0.62, 2.13)	1.05 (0.82, 1.35)	**0.41 (0.17, 0.99)**	2.67 (0.35, 5.29)	0.30 (0.07, 1.17)
BMI (per 1 kg/m^2^ increase)	1.02 (0.99, 1.05)	**0.48 (0.40, 0.58)**	0.97 (0.94, 1.01)	**0.67 (0.57, 0.78)**	0.83 (0.64, 1.01)	**1.17 (1.04, 1.32)**

*Notes*: ADL = activity of daily living; BMI = body mass index; cognition score and the number of chronic diseases were regarded as time-varying variables in the model. The reference categories were defined as participants stable in no sarcopenia, probable sarcopenia, and sarcopenia status. Bold values indicated statistically significant (*p*-value < .05).

### Sensitivity Analysis

After including participants with loss to follow-up, the estimated transition intensities and probabilities were stable across sarcopenia status, indicating results were robust (Supplementary Table 5). In comparison with females, male participants have greater death probabilities regardless of their initial states. The probability of maintaining a sarcopenia-free state was greater for males at year 1. Moreover, males were less likely to transition from no sarcopenia to possible sarcopenia and more likely to reverse from the sarcopenia state (Supplementary Table 6). No significant difference in influencing factors through the transitions was found between male and female older adults (Supplementary Tables 7 and 8).

## Discussion

Evidence of transitions between sarcopenia states is rare, especially for Chinese older adults. As we know, this is the first study to examine the sarcopenia transitions and influencing factors among Chinese community-dwelling older adults. Our study used national longitudinal data and revealed that sarcopenia was a 2-way dynamic process with both progression and reversion across sarcopenia states.

A main finding of the present study was that older adults were more likely to transition to the possible sarcopenia state from the sarcopenia-free state. Meanwhile, the probability of reversing to no sarcopenia was greater among possible sarcopenic older adults, compared with sarcopenic participants. The Health, Aging, and Body Composition (Health ABC) study ascertained that presarcopenic individuals had the greatest probability of progression from no sarcopenia and reverting to the state of no sarcopenia (12). Swedish National Study on Aging and Care in Kungsholmen (SNAC-K) also revealed a greater 1-year transition probability between sarcopenia-free and sarcopenia state during 12-year follow-up (13). Although there was a discrepancy among presarcopenia (low muscle mass), probable sarcopenia (low muscle strength) and possible sarcopenia, consistent results indicated that possible sarcopenia, an intermediate state between sarcopenia-free and sarcopenia, should be considered as a critical time window to prevent sarcopenia progression and to promote its rehabilitation. Moreover, a particularly interesting result we found was that the probability of reversion to sarcopenia-free was much higher than progressing to sarcopenia among possible sarcopenic participants. In contrast with this study, Trevisan et al. found that the 1-year probability of sarcopenia (6.7%) was higher than that of reverting to no sarcopenia (3.9%) for probable sarcopenic older adults. However, the 10-year probability of reverting increased and exceeded the likelihood of progressing to sarcopenia (13). A key difference between these 2 studies might be attributed to the inconsistent diagnostic algorithm of the intermediate sarcopenia state, namely probable and possible sarcopenia. In comparison with probable sarcopenia proposed by EWGSOP 2, possible sarcopenia proposed by the AWGS 2019 took indicators of muscle strength and physical performance, easily obtained in primary health care or community preventive services settings, as a priority consideration to enable early lifestyle interventions in diet and exercise (11). Considering low physical performance as an additional assessment item for possible sarcopenia, the prevalence of the intermediate sarcopenia state might be higher using the AWGS 2019 criteria. In the present study, 35.7% of participants were identified with possible sarcopenia, which was higher than the prevalence of probable sarcopenia (27.0%) reported by Trevisan et al. (13) And 89.2% of possible sarcopenic older adults had low physical performance, which could be associated with a greater reverse transition probability. No obvious symptom was shown in the early stage of sarcopenia. Sarcopenic older adults did not recognize their conditions until an extreme decline in muscle function (33). Our findings further shed light on the importance of identifying individuals with possible sarcopenia and implementing effective interventions in this critical stage.

Another main finding in our study was the influencing factors related to sarcopenia progression and reversion, which could guide the tailored prevention and intervention of sarcopenia for Chinese older residents. As for the influencing factors of sarcopenia progression, we found that advanced age, rural living, higher chronic disease numbers, and noncurrent drinking habits were positively associated with sarcopenia progression. Recent evidence suggested that underlying sarcopenia mechanisms were the age-accelerated unbalance of protein metabolism, involving chronic inflammation, oxidative stress, and alterations of mitochondrial function (34,35). Abundant literature also confirmed that advanced age was significantly associated with sarcopenia (23,36,37). Older Chinese adults living in rural areas were reported to be more vulnerable to be prevalent with sarcopenia, which could be interpreted as low muscle mass and strength caused by higher malnutrition risk (38). In combination with our results, additional attention should be paid to prevent sarcopenia progression among advanced older adults living in rural areas of China. In addition, the sarcopenia prevalence was reported to be higher among people with long-term medical conditions (39,40). Our results further revealed that chronic disease numbers might be related to an increasing risk of sarcopenia progression. Alcohol consumption has been identified as a lifestyle-related sarcopenia risk factor (2,37). On the contrary, the present result showed that current drinkers had a less progression probability. This finding could be linked to the fact that current drinkers might be under a healthier status compared to never or former drinkers, thus preventing the development of sarcopenia. However, it is still essential to educate the public about keeping healthy lifestyles to prevent sarcopenia progression (41).

Moreover, cognition level and BMI status were found to be related to both progression and reversion in the present study. A recent metaanalysis indicated that the prevalence of mild cognitive impairment (MCI) was higher among people with sarcopenia (42). A longitudinal study conducted among Chinese also demonstrated sarcopenia status was associated with worse cognition levels (43). Our findings reconfirmed the relationship and indicated that improving cognition status for older adults with possible sarcopenia and sarcopenia might contribute to effective rehabilitation. Accumulating evidence has shown that nutritional status plays a significant role in the development of sarcopenia (44,45). Malnutrition and sarcopenia were reported to have shared components, with a low-inflammatory state being an important factor (46). BMI was considered to be a simple indicator to reflect the nutritional status of older adults. We observed that each 1-unit BMI increase was associated with a 52% and 33% lower probability of sarcopenia progression for no sarcopenia and possible sarcopenia in older individuals, respectively. An increase per unit of BMI was also associated with a 17% higher reversion possibility to the possible sarcopenia state. More research was needed to figure out how to implement tailored cognitive and nutritional interventions, in order to effectively delay the progression of sarcopenia and promote its recovery.

The present study had several strengths. First, the high quality of the CHARLS data from a nationally representative population strengthened our findings. Second, this is the first longitudinal study investigating both the transitions across different sarcopenia statuses and influencing factors among Chinese community-dwelling older adults. Third, a continuous-time multistate Markov model was performed to analyze the process of sarcopenia transitions. All sarcopenia states and temporal information about interstate transitions were considered to obtain a better understanding of sarcopenia transitions.

There are still several limitations in our study. First, we excluded older adults with missing sarcopenia at baseline and during follow-up. These participants were more likely to be under a worse health status in comparison with our analytic sample, which may introduce selection bias and limit the extrapolation of our conclusions. Second, enough transition frequencies across different sarcopenia stages were not observed due to the short follow up duration in the CHARLS, especially for the transitions from no sarcopenia (5.6%) and possible sarcopenia (4.5%) to sarcopenia. Thus, stratified analysis except for sex was not performed in this study and it was not possible to have a precise estimation of long-term sarcopenia transition. Further studies with larger sample sizes and longer follow up duration among Chinese older adults are needed to validate and improve our findings. Third, walking speed was measured by a 2.5-m walking test, rather than a 6-m walking test recommended by the AWGS 2019. However, as reported in a systematic review, walking speed was not influenced by the distance older adults walked (47). Wu et al. (23) also found that the gait speed measured by 2.5-m was in line with previous Chinese studies. Therefore, a 2.5-m distance might be appropriate for assessing walking speed among Chinese older adults. Fourth, although we have included covariates related to sarcopenia transitions, some potential covariates, such as physical activity and dietary intake, were not considered due to the large missing proportion. In addition, information on chronic diseases and habits of smoking and drinking were self-reported, which might have affected the results to some extent. However, self-reported data in the CHARLS have been widely used and shown to be valid in sarcopenia epidemiological studies (5,43,48). Finally, the sarcopenia assessment was not conducted continuously, and the exact time of transitions was unknown. However, constructing the continuous-time multistate Markov model through the package msm could effectively fit the panel data from the processes with arbitrary observation times and obtain stable results without exact time of transitions (28,30).

## Conclusions and Implications

In summary, we found that sarcopenia was a dynamic process among Chinese older adults. Possible sarcopenia status might be a critical time window to promote sarcopenia reversion. Participants with older age, rural living, and multimorbidity should be cared to prevent sarcopenia progression. Cognitive and nutritional interventions targeting healthy and sarcopenic older adults should receive equal attention. Longitudinal studies with larger sample sizes and longer follow up duration are needed to further refine our findings and provide tailored guidance about sarcopenia.

## Supplementary Material

igad105_suppl_Supplementary_Material
