# Supplementary material for: Sarcopenia Transitions and Influencing Factors Among Chinese Older Adults With Multistate Markov Model
Source: Innov Aging. 2023 Sep 20;7(8):igad105. doi: 10.1093/geroni/igad105 (PMC10637947; doi:10.1093/geroni/igad105)
Supplement: igad105_suppl_Supplementary_Material [file igad105_suppl_supplementary_material.docx]

**Online Supplementary Material**

Supplementary Figure S1 Alluvial plot of the sarcopenia transitions among the Chinese community-dwelling elders during approximately 4-year follow-up.

Supplementary Figure S2 The compliance of observed prevalence rates (blue lines) with expected prevalence rates (red lines) for each state. The consistency between two lines indicated a better model fit.

Supplementary Table S1 Comparisons of baseline characteristics between analytic sample and excluded older adults with missing sarcopenia information.

Supplementary Table S2 Mean sojourn time (years) in each state.

Supplementary Table S3 Univariate analysis of factors associated with transition from no sarcopenia, possible sarcopenia, and sarcopenia (*HR* (95% *CI*)).

Supplementary Table S4 Multivariate analysis of factors associated with transitions to death (*HR* (95% *CI*)).

Supplementary Table S5 Estimated transition intensities and 1-year probabilities for the multi-state Markov model after including loss to follow-up population (n=3146).

Supplementary Table S6 Estimated transition intensities and 1-year probabilities for the multi-state Markov model stratified by sex.

Supplementary Table S7 Multivariate analysis of factors associated with sarcopenia transitions (*HR* (95% *CI*)) for male participants.

Supplementary Table S8 Multivariate analysis of factors associated with sarcopenia transitions (*HR* (95% *CI*)) for female participants.

Supplemental Table S1 Comparisons of baseline characteristics between analytic sample and excluded older adults with missing sarcopenia information.

| Characteristics | Analytic sample (n=2856) | Excluded sample (n=4144) | *P* |
| --- | --- | --- | --- |
| **Sociodemographic factors** |  |  |  |
| Age (Years), mean (*SD*) ^a^ | 67.6 (6.1) | 68.8 (7.3) | <0.001 |
| 60-74 years old, n (%) ^b^ | 2416(84.6) | 3229(77.9) | <0.001 |
| Female, n (%) ^b^ | 1383 (48.4) | 2097 (50.6) | 0.077 |
| Rural-living, n (%) ^b^ | 1992 (69.7) | 2294 (55.4) | <0.001 |
| Elementary school or above, n (%) ^b^ | 1177 (41.2) | 1782 (43.0) | 0.143 |
| **Health-related factors** |  |  |  |
| ADL difficulty, n (%) ^b^ | 570 (20.1) | 1063 (26.2) | <0.001 |
| Cognition score, mean (*SD*) ^a^ | 12.6 (5.7) | 12.4 (6.2) | 0.245 |
| Number of chronic diseases, median (*IQR*) | 1.0(0.0,2.0) | 1.0(1.0,2.0) | 0.194 |
| **Behavior-risk factors** |  |  |  |
| Current smokers, n (%) ^b^ | 937 (33.0) | 1030 (26.3) | <0.001 |
| Current drinkers, n (%) ^b^ | 683 (25.6) | 928 (24.4) | 0.303 |
| BMI (kg/m^2^), mean (*SD*) ^a^ | 22.7 (3.8) | 23.0 (4.2) | 0.002 |

Notes: ^a^ These variables were compared using two independent sample *t* test; ^b^ These variables were compared using chi-square test; ^c^ These variables were compared using Mann-Whitney *U* test; ADL=activity of daily living; BMI=body mass index; SD=standard deviation; IQR=interquartile range.


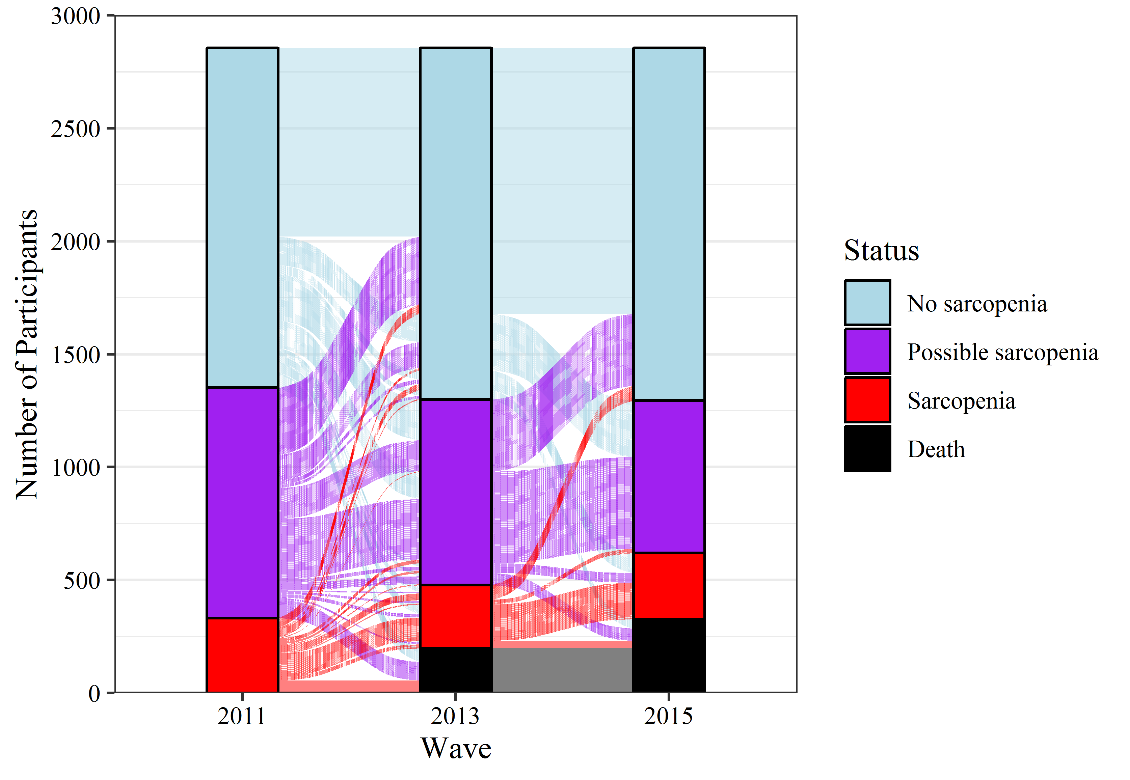
Supplementary Figure S1 Alluvial plot of the sarcopenia transitions among the Chinese community-dwelling elders during approximately 4-year follow-up.


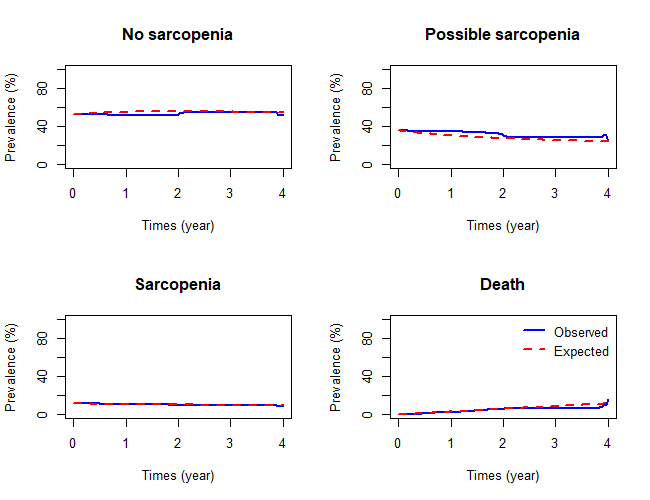


Supplementary Figure S2 The compliance of observed prevalence rates (blue lines) with expected prevalence rates (red lines) for each state. The consistency between two lines indicated a better model fit.

Supplementary Table S2 Mean sojourn time (years) in each state.

| Status | Sojourn time | 95% *CI* |
| --- | --- | --- |
| Initial status | - | - |
| No sarcopenia | 4.34 | 3.99-4.70 |
| Possible sarcopenia | 2.17 | 2.00-2.33 |
| Sarcopenia | 2.94 | 2.60-3.27 |

Supplementary Table S3 Univariate analysis of factors associated with sarcopenia transitions (*HR* (95% *CI*)).

| Characteristics | From no sarcopenia to | | |  | From possible sarcopenia to | | |  | From sarcopenia to | | |
| --- | --- | --- | --- | --- | --- | --- | --- | --- | --- | --- | --- |
|  | Possible sarcopenia | Sarcopenia | Death |  | No sarcopenia | Sarcopenia | Death |  | No sarcopenia | Possible sarcopenia | Death |
| **Sociodemographic factors** | | | | | | | | | | | |
| Age (ref=60-74) | **1.50(1.10,2.06)** | **4.90(3.15,7.58)** | 1.39(0.39,4.97) |  | **0.30(0.20,0.43)** | **4.65(2.02,10.71)** | **2.65(1.77,3.95)** |  | 0.69(0.45,1.07) | 0.98(0.28,3.51) | **4.72(2.78,8.00)** |
| Gender (ref=male) | 1.15(0.95,1.41) | 0.92(0.63,1.34) | 0.62(0.32,1.22) |  | 0.96(0.81,1.14) | 0.71(0.32,1.58) | **0.53(0.35,0.79)** |  | 0.93(0.63,1.37) | 0.77(0.27,2.15) | 0.83(0.53,1.29) |
| Region (ref=urban) | 1.14(0.92,1.42) | **2.47(1.44,4.23)** | 0.64(0.34,1.20) |  | 0.98(0.81,1.18) | 0.59(0.27,1.30) | 0.69(0.46,1.03) |  | 1.30(0.74,2.27) | 0.37(0.13,1.02) | 0.91(0.53,1.58) |
| Education (ref=lower than elementary school) | **0.77(0.63,0.94)** | 0.70(0.48,1.02) | 1.29(0.68,2.44) |  | **1.38(1.15,1.65)** | 0.19(0.02,1.81) | 1.24(0.83,1.85) |  | **2.27(1.53,3.38)** | 0.44(0.04,5.46) | 0.50(0.24,1.02) |
| **Health-related factors** | | | | | | | | | | | |
| ADL difficulty (ref=no) | 1.28(0.99,1.64) | 1.52(0.95,2.45) | 1.64(0.77,3.52) |  | **0.57(0.46,0.72)** | 1.05(0.44,2.50) | **1.57(1.05,2.34)** |  | 0.83(0.51,1.35) | 1.35(0.44,4.07) | 1.38(0.86,2.22) |
| Cognition score (per 1-point increase) | **1.10(1.03,1.17)** | 0.95(0.83,1.08) | 1.14(0.96,1.35) |  | **0.93(0.88,0.99)** | 0.94(0.74,1.20) | 1.11(0.99,1.23) |  | 0.89(0.76,1.03) | 1.09(0.82,1.45) | 1.03(0.89,1.18) |
| Number of chronic diseases | **0.97(0.95,0.98)** | **0.93(0.90,0.97)** | 0.97(0.92,1.02) |  | **1.05(1.03,1.06)** | **0.88(0.79,0.97)** | 1.01(0.97,1.05) |  | **1.05(1.01,1.09)** | **1.12(1.01,1.24)** | **0.90(0.85,0.95)** |
| **Behavior-risk factors** | | | | | | | | | | | |
| Smoking status (ref=non-current) | 0.88(0.71,1.09) | 1.15(0.78,1.72) | 1.78(0.95,3.33) |  | 1.04(0.86,1.26) | **2.96(1.32,6.63)** | 1.11(0.72,1.70) |  | 1.05(0.69,1.62) | **3.94(1.25,12.43)** | 0.91(0.56,1.49) |
| Drinking status (ref=non-current) | 0.98(0.79,1.21) | 0.94(0.62,1.40) | 1.57(0.84,2.93) |  | 1.19(0.98,1.45) | 0.70(0.23,2.14) | 1.15(0.74,1.77) |  | 1.47(0.96,2.24) | 0.79(0.19,3.32) | 0.78(0.44,1.37) |
| BMI (per 1 kg/m^2^ increase) | 1.01(0.97,1.04) | **0.64(0.54,0.76)** | 0.95(0.87,1.04) |  | 0.98(0.95,1.01) | **0.52(0.38,0.70)** | 0.98(0.93,1.04) |  | 0.97(0.86,1.08) | 1.01(0.83,1.23) | 0.95(0.85,1.05) |

ADL, activity of daily living; BMI, body mass index.

Cognition score and the number of chronic diseases were regarded as time-varying variables in the model. The reference categories were defined as participants stable in no sarcopenia, probable sarcopenia, and sarcopenia status. Bold values indicated statistically significant (*P*-value<0.05).

Supplementary Table S4 Multivariate analysis of factors associated with transitions to death (*HR* (95% *CI*)).

| Characteristics | From no sarcopenia |  | From possible sarcopenia |  | From sarcopenia |
| --- | --- | --- | --- | --- | --- |
| **Sociodemographic factors** | | | | | |
| Age (ref=60-74) | 0.74(0.12,4.54) |  | **3.12(1.99,4.88)** |  | **4.33(2.33,8.06)** |
| Gender (ref=male) | 0.70(0.32,1.55) |  | **0.54(0.32,0.93)** |  | **0.44(0.23,0.85)** |
| Region (ref=urban) | 0.59(0.33,1.04) |  | 0.71(0.46,1.11) |  | 0.75(0.41,1.38) |
| Education level (ref=lower than elementary school) | 1.28(0.64,2.53) |  | 1.15(0.69,1.92) |  | 0.59(0.22,1.56) |
| **Health-related factors** | | | | | |
| ADL difficulty (ref=no) | 1.78(0.92,3.47) |  | 1.54(0.98,2.42) |  | 1.38(0.81,2.37) |
| Cognition score (per 1-point increase) | 0.95(0.89,1.01) |  | 1.01(0.96,1.05) |  | 1.11(0.85,0.97) |
| Number of chronic diseases | **1.20(1.03,1.41)** |  | 1.11(0.99,1.25) |  | 0.95(0.81,1.12) |
| **Behavior-risk factors** | | | | | |
| Smoking status (ref=non-current) | 1.48(0.72,3.05) |  | 0.91(0.53,1.57) |  | 0.77(0.41,1.45) |
| Drinking status (ref=non-current) | 1.09(0.59,2.03) |  | 1.05(0.64,1.73) |  | 0.69(0.36,1.32) |
| BMI (per 1 kg/m^2^ increase) | 0.94(0.86,1.03) |  | 0.99(0.92,1.05) |  | 0.90(0.80,1.01) |

*Notes*: ADL=activity of daily living; BMI=body mass index; Cognition score and the number of chronic diseases were regarded as time-varying variables in the model. The reference categories were defined as participants stable in no sarcopenia, probable sarcopenia, and sarcopenia status. Bold values indicated statistically significant (*P*-value<0.05).

Supplementary Table S5 Estimated transition intensities and 1-year probabilities for the multi-state Markov model after including loss to follow-up population (n=3146).

| Transition | Intensities (95% *CI*) | 1-year probabilities (95% *CI*) |
| --- | --- | --- |
| **From no Sarcopenia** |  |  |
| No sarcopenia | - | 0.806(0.793, 0.818) |
| Possible sarcopenia | 0.179(0.163, 0.198) | 0.127(0.116, 0.137) |
| Sarcopenia | 0.042(0.035, 0.051) | 0.033(0.029, 0.039) |
| Loss to follow-up | 0.017(0.012, 0.022) | 0.018(0.015, 0.023) |
| Death | 0.012(0.009, 0.017) | 0.015(0.013, 0.019) |
| **From possible sarcopenia** |  |  |
| Possible sarcopenia | - | 0.636(0.617, 0.652) |
| No Sarcopenia | 0.375(0.344, 0.409) | 0.265(0.249, 0.283) |
| Sarcopenia | 0.029(0.019, 0.042) | 0.024(0.019, 0.032) |
| Loss to follow-up | 0.044(0.036, 0.054) | 0.038(0.032, 0.046) |
| Death | 0.042(0.035, 0.051) | 0.037(0.031, 0.043) |
| **From sarcopenia** |  |  |
| Sarcopenia | - | 0.701(0.668, 0.725) |
| No Sarcopenia | 0.195(0.161, 0.237) | 0.153(0.132, 0.180) |
| Possible sarcopenia | 0.054(0.032, 0.091) | 0.048(0.035, 0.071) |
| Loss to follow-up | 0.026(0.017, 0.041) | 0.024(0.017, 0.036) |
| Death | 0.085(0.068, 0.106) | 0.074(0.061, 0.089) |

Supplementary Table S6 Estimated transition intensities and 1-year probabilities for the multi-state Markov model stratified by sex.

| Transitions | Male | |  | Female | |
| --- | --- | --- | --- | --- | --- |
|  | Intensities (95% *CI*) | 1-year probabilities (95% *CI*) |  | Intensities (95% *CI*) | 1-year probabilities  (95% *CI*) |
| **From no Sarcopenia** |  |  |  |  |  |
| No sarcopenia | - | 0.828(0.811, 0.842) |  | - | 0.817(0.797, 0.834) |
| Possible sarcopenia | 0.164(0.143, 0.189) | 0.118(0.105, 0.132) |  | 0.190(0.165, 0.219) | 0.138(0.122, 0.156) |
| Sarcopenia | 0.044(0.034, 0.056) | 0.035(0.028, 0.043) |  | 0.040(0.030, 0.053) | 0.033(0.026, 0.042) |
| Death | 0.015(0.010, 0.022) | 0.020(0.015, 0.026) |  | 0.009(0.005, 0.016) | 0.012(0.009, 0.018) |
| **From possible sarcopenia** |  |  |  |  |  |
| Possible sarcopenia | - | 0.635(0.604, 0.660) |  | - | 0.671(0.644, 0.695) |
| No Sarcopenia | 0.397(0.349, 0.450) | 0.284(0.258, 0.312) |  | 0.382(0.338, 0.431) | 0.278(0.253, 0.304) |
| Sarcopenia | 0.034(0.020, 0.058) | 0.029(0.020, 0.043) |  | 0.024(0.013, 0.044) | 0.023(0.016, 0.036) |
| Death | 0.061(0.048, 0.078) | 0.053(0.043, 0.065) |  | 0.032(0.023, 0.044) | 0.029(0.022, 0.038) |
| **From sarcopenia** |  |  |  |  |  |
| Sarcopenia | - | 0.698(0.653, 0.734) |  | - | 0.731(0.684, 0.764) |
| No Sarcopenia | 0.206(0.156, 0.272) | 0.164(0.131, 0.205) |  | 0.191(0.145, 0.252) | 0.153(0.124, 0.193) |
| Possible sarcopenia | 0.062(0.031, 0.124) | 0.053(0.035, 0.088) |  | 0.047(0.021, 0.104) | 0.046(0.030, 0.084) |
| Death | 0.097(0.072, 0.132) | 0.085(0.065, 0.111) |  | 0.081(0.058, 0.111) | 0.071(0.052, 0.095) |

Supplementary Table S7 Multivariate analysis of factors associated with sarcopenia transitions (*HR* (95% *CI*)) for male participants.

| Characteristics | From no sarcopenia to | |  | From possible sarcopenia to | |  | From sarcopenia to | |
| --- | --- | --- | --- | --- | --- | --- | --- | --- |
|  | Possible sarcopenia | Sarcopenia |  | No sarcopenia | Sarcopenia |  | No sarcopenia | Possible sarcopenia |
| **Sociodemographic factors** | | | | | | | | |
| Age (ref=60-74) | 1.38(0.91,2.07**)** | **2.14(1.23,3.74)** |  | **0.31(0.19,0.50)** | **2.96(1.51,5.81)** |  | 0.71(0.40,1.28) | 0.82(0.35,1.94) |
| Region (ref=urban) | 1.11(0.84,1.47) | 1.47(0.85,2.56) |  | 1.10(0.85,1.42) | 0.66(0.30,1.43) |  | 0.80(0.45,1.42) | 1.09(0.43,2.81) |
| Education level (ref=lower than elementary school) | 0.85(0.65,1.12) | 0.75(0.45,1.24) |  | 1.04(0.82,1.33) | 0.82(0.38,1.77) |  | 1.67(0.98,2.83) | 0.76(0.32,1.79) |
| **Health-related factors** | | | | | | | | |
| ADL difficulty (ref=no) | 1.11(0.77,1.61) | 1.42(0.75,2.71) |  | **0.64(0.47,0.88)** | 1.10(0.47,2.53) |  | 0.83(0.42,1.66) | 0.57(0.19,1.70) |
| Cognition score (per 1-point increase) | 0.98(0.95,1.01) | 1.01(0.96,1.06) |  | **1.03(1.01,1.06)** | **0.92(0.86,0.99)** |  | 1.04(0.99,1.10) | 1.04(0.96,1.11) |
| Number of chronic diseases | **1.12(1.04,1.22)** | 1.01(0.85,1.20) |  | 0.96(0.89,1.03) | 1.02(0.84,1.25) |  | 0.83(0.67,1.03) | 0.97(0.76,1.38) |
| **Behavior-risk factors** | | | | | | | | |
| Smoking status (ref=non-current) | 0.99(0.77,1.28) | 0.85(0.53,1.38) |  | 0.96(0.76,1.21) | 1.45(0.69,3.05) |  | 1.23(0.72,2.10) | 1.40(0.60,3.26) |
| Drinking status (ref=non-current) | 1.19(0.93,1.53) | 0.90(0.56,1.43) |  | 1.26(0.99,1.58) | **0.45(0.21,0.96)** |  | 1.44(0.87,2.40) | 0.82(0.36,1.87) |
| BMI (per 1 kg/m^2^ increase) | 0.99(0.94,1.08) | **0.53(0.47,0.61)** |  | 0.98(0.94,1.02) | **0.63(0.53,0.74)** |  | 1.13(0.98,1.31) | 0.89(0.75,1.06) |

Notes: ADL=activity of daily living; BMI=body mass index; Cognition score and the number of chronic diseases were regarded as time-varying variables in the model. The reference categories were defined as participants stable in no sarcopenia, probable sarcopenia, and sarcopenia status. Bold values indicated statistically significant (*P*-value<0.05).

Supplementary Table S8 Multivariate analysis of factors associated with sarcopenia transitions (*HR* (95% *CI*) for female participants.

| Characteristics | From no sarcopenia to | |  | From possible sarcopenia to | |  | From sarcopenia to | |
| --- | --- | --- | --- | --- | --- | --- | --- | --- |
|  | Possible sarcopenia | Sarcopenia |  | No sarcopenia | Sarcopenia |  | No sarcopenia | Possible sarcopenia |
| **Sociodemographic factors** | | | | | | | | |
| Age (ref=60-74) | **1.84(1.15,2.95)** | **3.02(1.15,7.90)** |  | **0.29(0.16,0.52)** | 1.66(0.48,5.79) |  | 0.85(0.37,1.99) | 0.03(0.01,1.02) |
| Region (ref=urban) | 0.92(0.63,1.33) | 2.45(0.91,6.62) |  | 0.82(0.60,1.12) | 1.05(0.02,1.57) |  | 2.70(0.96,6.84) | **0.08(0.01,0.18)** |
| Education level (ref=lower than elementary school) | 0.72(0.48,1.08) | 1.56(0.64,3.81) |  | 0.94(0.68,1.31) | 0.10(0.03,26.89) |  | 1.74(0.67,4.54) | 0.05(0.11,1.65) |
| **Health-related factors** | | | | | | | | |
| ADL difficulty (ref=no) | 1.07(0.75,1.53) | 1.15(0.50,2.64) |  | **0.62(0.45,0.86)** | 2.94(0.65,11.32) |  | 0.89(0.43,1.87) | 3.42(0.17,6.83) |
| Cognition score (per 1-point increase) | 0.97(0.94,1.01) | 0.90(0.84,0.96) |  | **1.03(1.01,1.05)** | 1.00(0.89,1.13) |  | 1.03(0.97,1.10) | 1.70(1.13,2.57) |
| Number of chronic diseases | 1.09(0.99,1.20) | 1.00(0.79,1.25) |  | 0.96(0.87,1.04) | 1.26(0.88,1.81) |  | 0.95(0.70,1.19) | 0.87(0.35,1.72) |
| **Behavior-risk factors** | | | | | | | | |
| Smoking status (ref=non-current) | 0.79(0.44,1.42) | 0.15(0.04,1.60) |  | 0.72(0.44,1.16) | 1.62(0.48,8.85) |  | 1.19(0.04,1.95) | 3.27(0.15,5.62) |
| Drinking status (ref=non-current) | 0.82(0.50,1.36) | 5.20(0.03,29.23) |  | 0.73(0.45,1.19) | 0.67(0.08,5.47) |  | 1.49(0.01,8.41) | 0.13(0.03,3.87) |
| BMI (per 1 kg/m^2^ increase) | 1.05(0.98,1.09) | **0.56(0.46,0.68)** |  | 0.99(0.95,1.03) | 0.91(0.78,1.08) |  | 0.79(0.62,1.01) | **1.54(1.14,2.07)** |

Notes: ADL=activity of daily living; BMI=body mass index; Cognition score and the number of chronic diseases were regarded as time-varying variables in the model. The reference categories were defined as participants stable in no sarcopenia, probable sarcopenia, and sarcopenia status. Bold values indicated statistically significant (*P*-value<0.05).
